# Fusion of Ni Plating on CP-Titanium by Electron Beam Single-Track Scanning: Toward a New Approach for Fabricating TiNi Self-Healing Shape Memory Coating

**DOI:** 10.3390/ma16155449

**Published:** 2023-08-03

**Authors:** Lei Wang, Masayuki Okugawa, Hirokazu Konishi, Yuheng Liu, Yuichiro Koizumi, Takayoshi Nakano

**Affiliations:** 1 Graduate School of Engineering, Osaka University, 2-1 Yamadaoka, Suita 565-0871, Japan; lei.wang@mat.eng.osaka-u.ac.jp (L.W.); konishi@mat.eng.osaka-u.ac.jp (H.K.); yuheng.liu@mat.eng.osaka-u.ac.jp (Y.L.); nakano@mat.eng.osaka-u.ac.jp (T.N.); 2 Anisotropic Design & Additive Manufacturing Research Center, Osaka University, 2-1 Yamadaoka, Suita 565-0871, Japan

**Keywords:** commercially pure Ti, TiNi coating, shape memory alloy, pseudoelasticity, wear resistance, electron beam melting

## Abstract

The limited wear resistance of commercially pure titanium (CP-Ti) hinders its use in abrasive and erosive environments, despite its good strength–weight ratio and corrosion resistance. This paper reports the first study proposing a novel method for wear-resistant TiNi coating through Ni plating and electron beam (EB) irradiation in an in situ synthetic approach. Single-track melting experiments were conducted using the EB to investigate the feasibility of forming a TiNi phase by fusing the Ni plate with the CP-Ti substrate. Varying beam powers were employed at a fixed scanning speed to determine the optimal conditions for TiNi phase formation. The concentration of the melt region was found to be approximate as estimated from the ratio of the Ni-plate thickness to the depth of the melt region, and the region with Ni-48.7 at.% Ti was successfully formed by EB irradiation. The study suggests that the mixing of Ti atoms and Ni atoms was facilitated by fluid flow induced by Marangoni and thermal convections. It is proposed that a more uniform TiNi layer can be achieved through multi-track melting under appropriate conditions. This research demonstrates the feasibility of utilizing EB additive manufacturing as a coating method and the potential for developing TiNi coatings with shape memory effects and pseudoelasticity.

## 1. Introduction

Commercially pure titanium (CP-Ti) is widely used in biomedical [1], aerospace [2], chemical [3], marine [4], and nuclear industries [5] owing to its good corrosion resistance, biocompatibility, low weight, and high specific strength [6]. However, the poor wear resistance of the CP-Ti limits its mechanical performance [7,8] and its use in transmission components, such as engines and pumps.

TiNi alloy shows a good tolerance of the material to deformation because of its shape memory effect and pseudoelasticity, which can be observed even in TiNi alloy thin films [9]. Therefore, the use of TiNi alloy coating is expected to address the low wear resistance of CP-Ti. In addition, the TiNi coating is expected to impart self-healing properties to materials owing to its shape memory effect and pseudoelasticity [10,11]. Therefore, several studies have been conducted on TiNi coatings using traditional coating methods such as laser–plasma spraying [12,13], vacuum plasma spraying (VPS) [14,15], atmospheric plasma spraying (APS) [16], cold spraying [17,18,19], and magnetron sputtering [20,21]. The previous studies reported that the TiNi alloy coatings were successfully obtained by conventional methods. However, there is inhomogeneity in the microstructure: compositions and phases are different spatially on the order of 10 μm [13]. In addition, unfused regions and oxides are frequently observed in the coatings [15,16]. These defects must be removed to improve shape memory and pseudoelastic properties.

To overcome the disadvantages of the traditional coating method, we propose a novel method to form TiNi coatings using the electron beam (EB) melting method, which is a technique used in welding and additive manufacturing (AM). A TiNi alloy layer is synthesized by EB irradiation to the electroplated pure Ni top surface layer and mixing it with part of the underlying CP-Ti substrate. A sturdy and strongly bonded coating can be obtained because it eliminates the need to fill the gap between the base and coating materials to be bonded in contrast to spray coating methods. Moreover, the composition and microstructure can be adjusted by tuning the EB parameters, which can help obtain shape memory, pseudoelasticity, and self-healing features. However, the optimum conditions and appropriate spatial distribution of solute atoms and crystal orientation must be determined to obtain a coating with the desired properties. 

In this study, we fabricated TiNi coatings with various EB parameters to determine the optimum process parameters. The relationship between the process parameters and coating quality, including the compositional uniformity and surface roughness, is discussed in view of the various phenomena such as melting, stirring, and solidification.

## 2. Materials and Methods

A slab of CP-Ti (Nilaco, Tokyo, Japan, Purity 99.5%) was cut into specimens with 20 mm × 20 mm × 10 mm using electric discharge machining. One of the 20 mm × 20 mm surfaces was coated with Ni via electrochemical plating, using nickel sulfamate solution as the plating solution. The specimen was immersed in the plating solution, held at a 0.10 A cathodic current limit and 4.00 V voltage limit for 2 h. The compositions of the solutions and the plating parameters are listed in Table 1.

The plated specimens were irradiated with EB and scanned along 4 mm long straight lines using an EB machine (Mitsubishi Electric, EBM-6LB-1) under a helium atmosphere at 0.5 Pa. The acceleration voltage and convergence current were fixed at 60 kV and 1150 mA, respectively. The irradiations were conducted under six levels of beam power, *P*: 100, 200, 300, 400, 500, and 600 W (i.e., beam currents, *I*: 1.67, 3.33, 5.00, 6.67, 8.33, and 10 mA, respectively), and a scanning speed of 100 mm s^−1^.

The top surfaces of the EB-irradiated samples were observed using a laser microscope (Keyence, Osaka, Japan, VK-X200/210) to measure the widths and surface topographies of the melt tracks. A scanning electron microscope (Keyence, VE-8800) equipped with energy dispersive X-ray spectroscopy was used to analyze the elemental composition of the melt tracks on the surface. Furthermore, the samples were cut perpendicular to the beam-scanning direction for cross-sectional observations using field-emission scanning electron microscopy (FE-SEM, JEOL, JSM 6500). In addition, Vickers harnesses of the melt regions were measured using a Vickers hardness tester (Shimadzsu, Kyoto, Japan, HMV-G31) with a 200 g load for 15 s. The test was conducted seven times for the cross sections of each sample, and the results were averaged. 

To discuss the formation process of the observed phases resulting from the dissolution of Ti from the substrate into the Ni melt formed by melting the Ni plate, we need to consider Ti as the solute and Ni as the solvent. Typically, TiNi phase diagrams are drawn with Ni concentration as the horizontal axis. However, for our analysis, we drew the equilibrium phase diagrams of the TiNi binary system using Ti concentration as the horizontal axis. To create these diagrams, we used Thermodynamic calculation software (Thermo-Calc Software AB, Thermo-Calc v2022b) and relied on the thermodynamic database for Titanium alloys (TCTI4). While Ti is treated as the solute, we chose to designate the phases with Ti followed by Ni (as in TiNi and Ti_2_Ni), for convenience in our discussion. 

## 3. Results

### 3.1. Surface Morphology of the Melt Track

**Figure 1:**
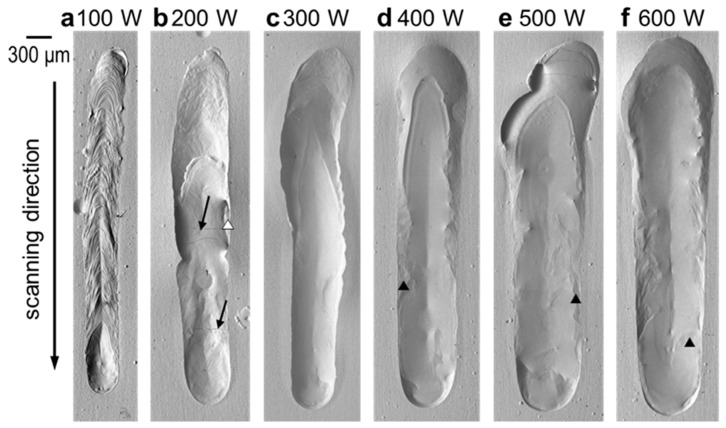
SEM images of the melt track surfaces formed by EB scanning with a constant scanning speed of 100 mm/s and various beam powers of (**a**) 100 W, (**b**) 200 W, (**c**) 300 W, (**d**) 400 W, (**e**) 500 W, and (**f**) 600 W. The arrows, white arrowheads, and black arrowheads indicate the cracks, the pits, and irregular patterns, respectively.

**Figure 2:**
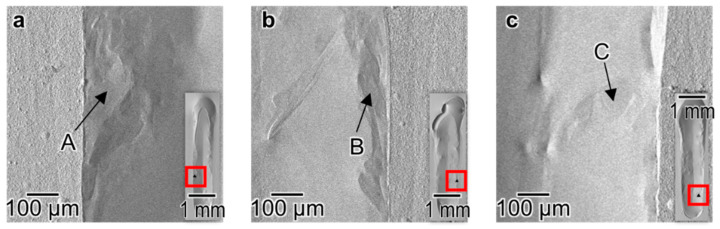
Magnified SEM images of the area with irregular patterns indicated by the red squares on the surface of the tracks formed by EB irradiation with the beam power of (**a**) 400 W, (**b**) 500 W, (**c**) 600 W, and a scanning speed of 100 mm/s. The A, B, and C indicated by the arrows are the points analyzed by SEM-EDS (Table 2, Figure S1).

Figure 1 shows SEM images of the melt track surfaces after EB scanning, where the EB was scanned downward. The surface topography of the melt track is influenced by the value of *P* [W]. At *P* of 100 W (Figure 1a), the melt track formed a chevron-like patterned surface, which did not appear at higher beam powers. This implies that solidification occurred immediately after melting when the surface topography was still significantly affected by the wave generated by the irradiation of the moving EB and moving melt pool. In addition, a teardrop-shaped concave surface follows from the shape of the melt pool. At 200 W (Figure 1b), the surface of the melt region was smooth compared with that at 100 W. However, cracks were observed, as indicated by the arrows. Moreover, holes were occasionally observed, as indicated by the white arrowheads. Li et al. [23] and Takase et al. [24] reported that the residual stresses up to hundreds of MPa are caused via melting and solidification by the EB irradiation of the AM process. It is supposed that the residual stresses are also caused by the EB irradiation in this study and lead to cracks near the melt–pool boundary of the tracks. The smoothest surface was obtained at 300 W (Figure 1c). It is worth noting that some irregular patterns distributed along the middle edges of the tracks formed by 400–600 W EB irradiation (Figure 1d–f), as indicated by black arrowheads and the magnified images, are shown in Figure 2. The EDS analysis revealed that the patterns consist of almost 100 at.% Ti, as shown in Table 2. 

**Figure 3:**
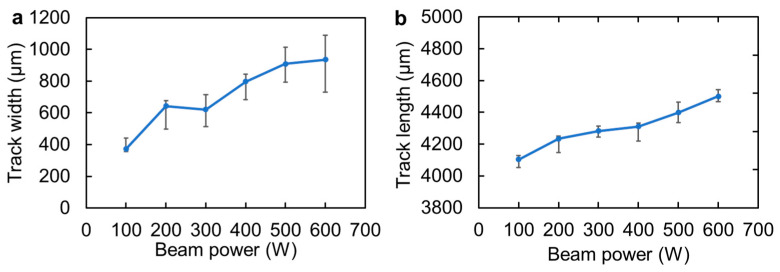
(**a**) Width and (**b**) length of the melt track formed by EB scanning as a function of the beam power. The scanning speed is 100 mm/s.

The widths and lengths of the melt tracks were measured and plotted against the beam power, as shown in Figure 3. The width significantly increased with an increase in *P* from 100 to 200 W, but was almost the same from 200 to 300 W. Moreover, the width increased significantly again from 300 to 400 W. The width gradually increased and saturated above 500 W. On the other hand, the length exhibited a monotonic and gradual increase with increasing *P* without a tendency to saturate. 

Miyata et al. [25] evaluated the shape of melt pools in stainless steel formed by single-track EB scanning with various *P* conditions and found that the width and height increased as the *P* increased. The trend between the shape of the melt track and the *P* is the same as that in the Ni-plated CP-Ti observed in this study. Miyata et al. [25] also reported that the change in the width can be reproduced by computational thermal–fluid dynamics using single absorption rates of 0.8. It is suggested that the difference in the shape of the melt track with the *P* is caused by the difference in the total amount of absorbed heat.

### 3.2. Cross-Sectional Observation of Melt Track

**Figure 4:**
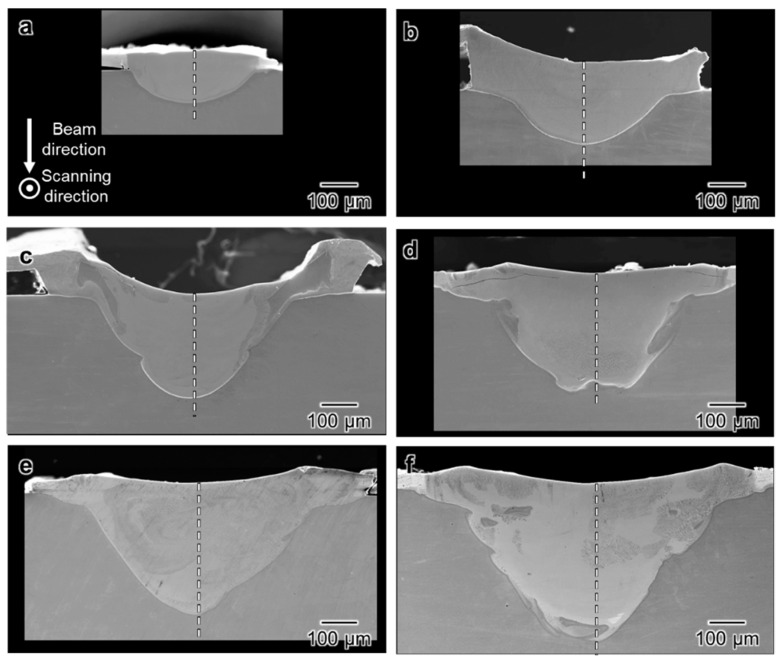
SEM images of the cross section of the melt track after EB scanning at a constant scanning speed of 100 mm/s and various levels of beam power (*P*): (**a**) 100 W; (**b**) 200 W; (**c**) 300 W; (**d**) 400 W; (**e**) 500 W; and (**f**) 600 W.

Figure 4a–f show the cross-sectional SEM images of the melt tracks corresponding to the top-view SEM images in Figure 1a–f. The areas of the melted regions increased with increasing EB power, and the wider and deeper melt track is formed under the high-power EB irradiation. However, the melt track was not equally enlarged in every dimension and the curvatures of the fusion lines are varied by *P*: For *P* = 100 W (Figure 4a) and 200 W (Figure 4b), the width of the melt track was much greater than the depth, and the fusion lines appeared to be elliptical. With an increase in beam power from 300 to 600 W (Figure 4c–f), the depth of the melt track increased more significantly than its width. As a result, the cross section of the melt track evolved into a triangle-like shape. In addition, the fusion line appeared irregularly bent and blurred under these conditions. 

**Figure 5:**
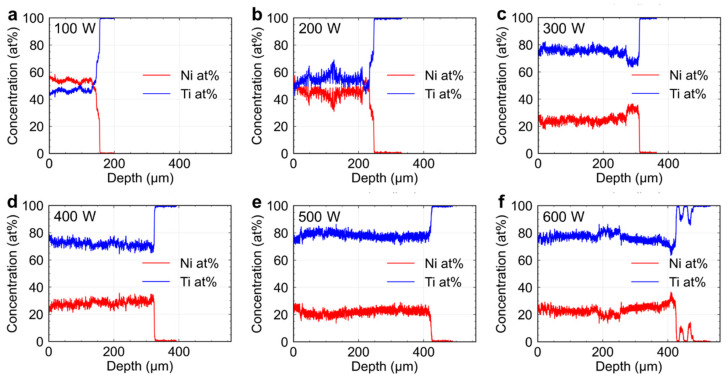
Elemental distributions analyzed by SEM-EDS at the center of the melt pools along the dashed line indicated in Figure 3: (**a**) 100 W; (**b**) 200 W; (**c**) 300 W; (**d**) 400 W; (**e**) 500 W; and (**f**) 600 W.

The thickness of the electroplated Ni layer was less than 50 μm (approximately 44 μm on average), and the edges of the melt region for *P* = 200 W (Figure 4b) and 300 W (Figure 4c) were much thicker than the original Ni layer. In particular, the electroplated Ni layers beside the melt region detached from the Ti substrate, as shown in Figure 4c. Moreover, the electroplated Ni layer is separated from the melt region on the right-hand side. Hydrogen inevitably emerges during the Ni-plating process [26] and some of the hydrogens trapped at the interface between the plated layer and the substrate, as well as within the plated layer, cause the formation of pores [27]. It has been reported that the pores weaken the bonding between the plating and the substrate [28], and cause localized separation of the plating from the substrate [29]. In this study, the pores are supposed to also be formed during the Ni plating and lead to causing the pits (Figure 2b) and the plating to bulge (Figure 4c,d). The separation of the electroplated Ni layer is also shown in Figure 4a. However, the effect of the separation distance for *P* = 100 W was not significant. The space can be filled by a subsequent EB scanning along an adjacent line with an interface of 200–300 μm in forming the alloy layer on the entire surface, which will be presented in a future report. Figure 5a–f show the EDS line profiles of the melt pools indicated by dashed lines in Figure 4. The atomic ratio of Ti:Ni is approximately 1:1, which is close to the stoichiometric chemical composition of the TiNi alloy in the melt regions formed by EB irradiation with *P* = 100 and 200 W, as shown in Figure 5a,b. On the other hand, the concentration distribution is not completely homogenous. Notably, the Ti atomic concentration at a distance of approximately 120 μm from the surface for *P* = 200 W even approaches 70%. This significant variation suggests local segregation within the melt track with *P* = 100 W and 200 W. The ratios of Ti:Ni in the melt regions formed with *P* = 300–600 W (Figure 5c–f) are approximately 3:1 with a more uniform special distribution. It is suggested that the time from the melting to the solidification is longer with a higher EB power and enough convection and diffusion of the Ni and Ti atoms are allowed to form the homogeneous melt pool. 

**Figure 6:**
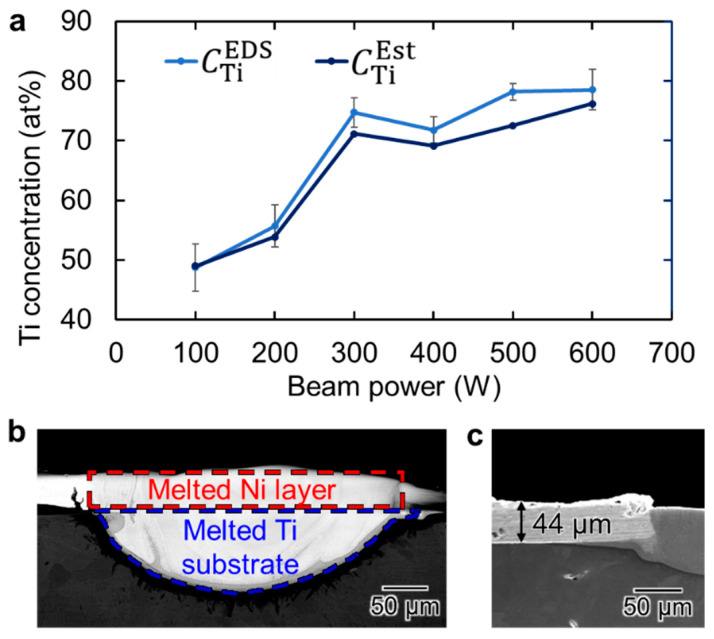
(**a**) Ti concentration as a function of EB power; (**b**) schematic of the area of the melted Ni layer and CP-Ti substrate; (**c**) cross-sectional SEM image of the Ni-plating layer.

The average Ti concentrations of the melt pools formed by various EB powers were calculated from the EDS line profiles (hereafter referred to as $C_{Ti}^{EDS}$), shown in Figure 6a as a function of *P*. The Ti concentration at 100 W was approximately 50 at.% Ti, which is almost the stoichiometric composition of TiNi. The Ti concentration increased with the beam power. The increase in Ti concentration is not linear, but increases abruptly and then slows down, gradually approaching 80%.

Figure 6b shows a sample cross-sectional SEM image of the melt region formed by EB irradiation at *P* = 100 W. The area enclosed by the red dotted line is the melted region of the electroplated Ni layer while the area enclosed by the blue dotted line is the melted region of the CP-Ti substrate. To analyze the distribution of elemental compositions in the melt track, we calculated the theoretical Ti atomic concentrations ($C_{Ti}^{Est}$) using the areas of the Ni and Ti melting regions for each melt track in the cross section. First, the differential volume of the melting metal ∆*V* at the position of the plane shown in the cross-section image can be expressed by the following equation:

∆*V*_Ti_ = *A*_Ti_ ∆*L* (1)

∆*V*_Ni_ = *A*_Ni_ ∆*L* (2)

where *A*_Ti_ is the area of the Ti melting region in the cross section, *A*_Ni_ is the area of the Ni melting region in the cross section, and ∆*L* is the differential track length. Moreover, *A*_Ti_ can be obtained by directly measuring the area of the Ti-melted region in the cross-sectional image. *A*_Ni_ could not be measured directly in the cross-sectional image because the melt track surface did not maintain its shape before irradiation. However, the electroplated Ni layer is thin and uniform; therefore, the estimated value of *A*_Ni_ can be obtained by multiplying the melt track width by the thickness of the electroplated Ni layer. An electroplated Ni layer that was not irradiated with EB can also be observed in Figure 6c. The thickness of the Ni layer was measured to be 44 μm as indicated by the double-headed arrow.

The differentiation of the amount of substance of melting metal ∆n can be calculated using the volume differentiation.

(3) $$∆n_{Ti}=\frac{{∆V_{Ti}\rho }_{Ti}}{M_{Ti}}$$

(4) $$∆n_{Ni}=\frac{∆V_{Ni}\rho_{Ni}}{M_{Ni}}$$

here, *ρ*_Ti_ and *ρ*_Ni_ are the densities of Ti and Ni, and *M*_Ti_ and *M*_Ni_ are the molar masses of Ti and Ni, respectively. Finally, the estimated Ti atomic concentration ($C_{Ti}^{Est}$) at the position of the plane shown in the cross-sectional image can be calculated using the following equation:

(5) $$C_{Ti}^{Est}=\frac{∆n_{Ti}}{∆n_{Ti}+∆n_{Ni}}\times 100\%$$

comparing the $C_{Ti}^{Est}$ with the $C_{Ti}^{EDS}$ (Figure 6a), both increased abruptly and then gradually approached 80%.

The $C_{Ti}^{Est}$ and $C_{Ti}^{EDS}$ of the melt pools formed by EB irradiations with *P* = 100 W and 200 W were very close to each other. On the other hand, $C_{Ti}^{Est}$ became smaller than $C_{Ti}^{EDS}$ of the melt pools formed by higher power EB irradiations. It is supposed that the surface temperature of the specimen exceeds the boiling point of Ni by the high-power EB irradiation and evaporation of Ni leads to an increase in the Ti concentrations in the melt pools.

**Figure 7:**
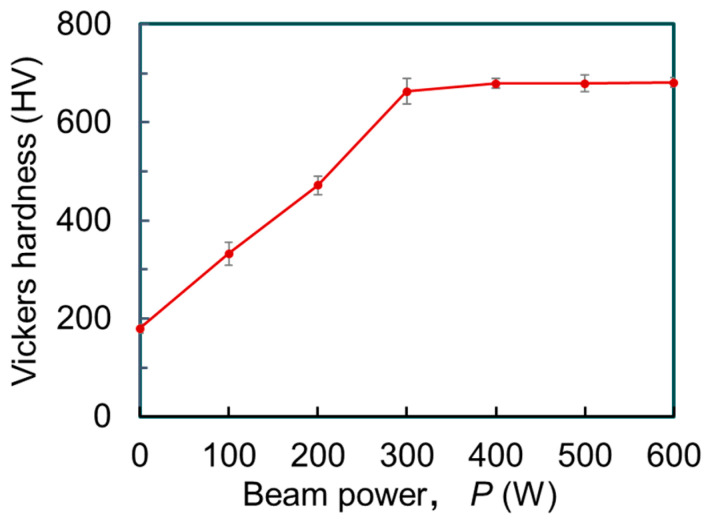
Vickers hardness of melt track after EB scanning plotted as a function of *P*, in the range from 0 W to 600 W. The point with *P* = 0 W represents the hardness of the CP-Ti substrate.

Vickers hardness of the melt tracks formed by EB irradiation with various *P* are shown in Figure 7, compared with the hardness of the CP-Ti substrate. The Vickers hardness increases monotonically with increasing *P*, approaching 680 HV. The TiNi layer, formed under all irradiation conditions, exhibits higher hardness than that of the CP-Ti substrate. The surface mechanical properties are expected to be improved by applying the EB irradiation method to the fabrication of TiNi alloy coatings on CP-Ti.

### 3.3. Spatial Elemental Distributions in the Melt Regions

Further detailed elemental distribution analysis was performed on the melted regions formed by the irradiation of EB with *P* = 100 and 200 W, in which the compositions are close to that of the TiNi phase, while these formed with *P* = 300 and 600 W, of which the melted regions show homogeneous concentration distributions in EDS profiles. 

**Figure 8:**
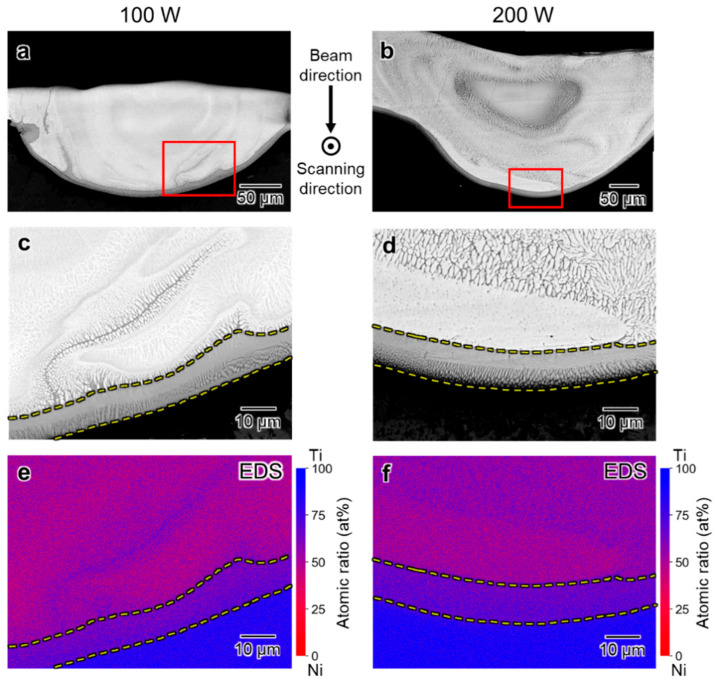
(**a**,**b**) Cross-sectional BSE images of the melt track at 100 and 200 W beam intensities; (**c**,**d**) magnified images of the region including a part of fusion lines indicated by a red box in (**a**,**b**); (**e**,**f**) Ti and Ni elemental maps of the region near the fusion line.

Figure 8a,b show the cross-sectional SEM back-scattered electron (BSE) images of the melt region for *P* = 100 W and 200 W, respectively. Significant variations in brightness within the melted areas are evident, with a notably darker layer appearing at the outermost periphery. The relative brightness of the BSE image is positively correlated with the atomic weight of the elements in the local area. The observed brightness fluctuations are suggested to reflect the fluctuated distribution of elements within the melt pools. Figure 8c,d show magnified images of the areas indicated by the red rectangular frames, respectively. The regions consist of a fine dendrite microstructure in both melt pools. The dendrite microstructure in the melt pool formed by EB irradiation with *P* = 100 W is finer than that with *P* = 200 W, and the widths are 0.9 μm and 1.2 μm, respectively. Miyata et al. [25] reported that the cooling rate of the regions melted by EB irradiation for the AM process is higher with a smaller EB power condition. It is suggested that the higher cooling rate in the 100 W condition causes the finer microstructure in this study. 

**Figure 9:**
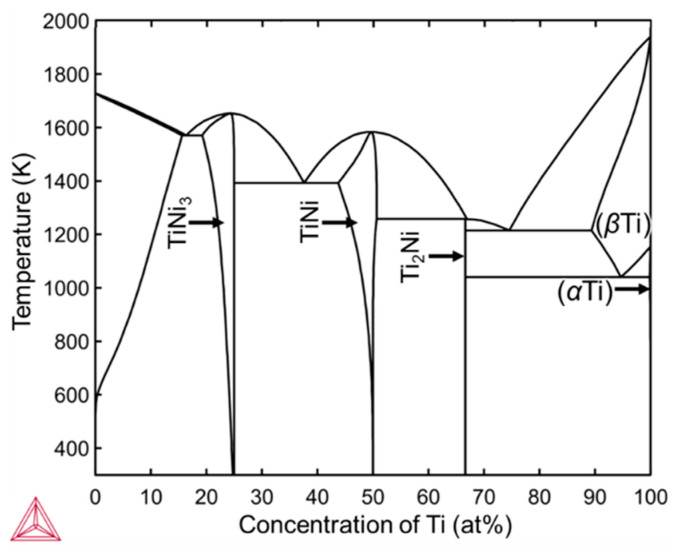
TiNi binary equilibrium phase diagram with concentration of Ti as horizontal axis calculated using the thermodynamic database for Titanium alloys (TCTI4).

Figure 8e,f show the EDS elemental maps corresponding to the BSE images in Figure 8c,d, respectively. The Ti concentration in the outermost layer is higher than that in the inner layer in both melt pools. Combining the BSE images (Figure 8c,d) and EDS mapping data (Figure 8e,f) and referring to the TiNi binary equilibrium phase diagram (Figure 9) [30], the outermost layers formed with *P* = 100 and 200 W are in the Ti_2_Ni phase, of which the Ti concentrations are 66.1–88.8 at.% Ti in the equilibrium state. On the other hand, the inner area of the melt track consists of the TiNi phase, of which the Ti concentrations are 46.4–54.4 at.% Ti in the equilibrium state.

**Figure 10:**
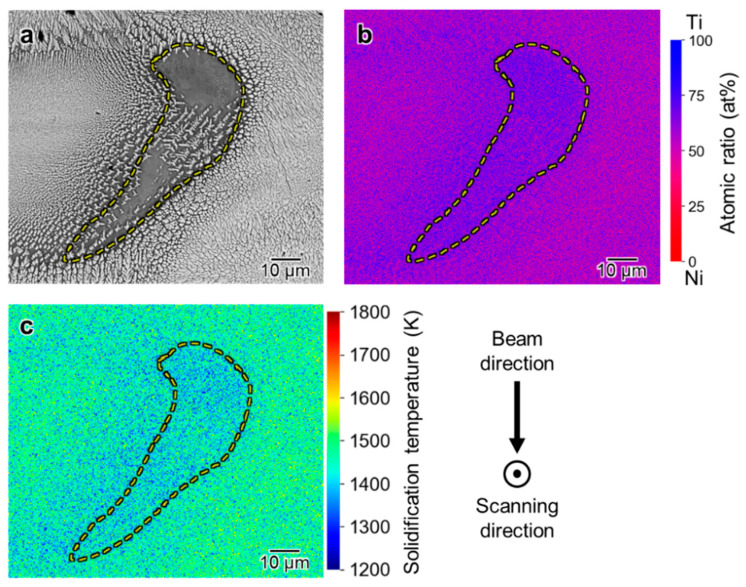
(**a**) Cross-sectional BSE image at the center of the melt track at 200 W beam intensity; (**b**) Ti and Ni atomic percentage mapping of the region in (**a**); (**c**) solidification-point mapping of the region in (**a**).

The contrast of the center part of the melt pools is different: the center part of the melt track formed by EB irradiation with *P* = 100 W shows a uniform bright contrast region, whereas that formed with *P* = 200 W has a dark contrasted region. Figure 10a,b shows the BSE and corresponding EDS elemental distribution maps of the dark phase at the center part of the melt track with *P* = 200 W. The EDS analysis revealed that the dark phase in the central part had a Ti atomic percentage of approximately 65%, which indicated that it is the Ti_2_Ni phase, as well as a dark phase appearing on the fusion line. The liquidus temperatures of the local regions are calculated using the compositions and the Ni-Ti binary phase diagram, as shown in Figure 10c, for considering the formation mechanism of the Ti_2_Ni phase. The liquidus temperature in the inner area of the yellow dashed line is significantly lower than that in the outer area. The Ti_2_Ni regions that appeared only in the melt track formed by EB irradiation at *P* = 200 W are suggested to be precipitated finally in the solidification process due to its low liquidus temperature.

**Figure 11:**
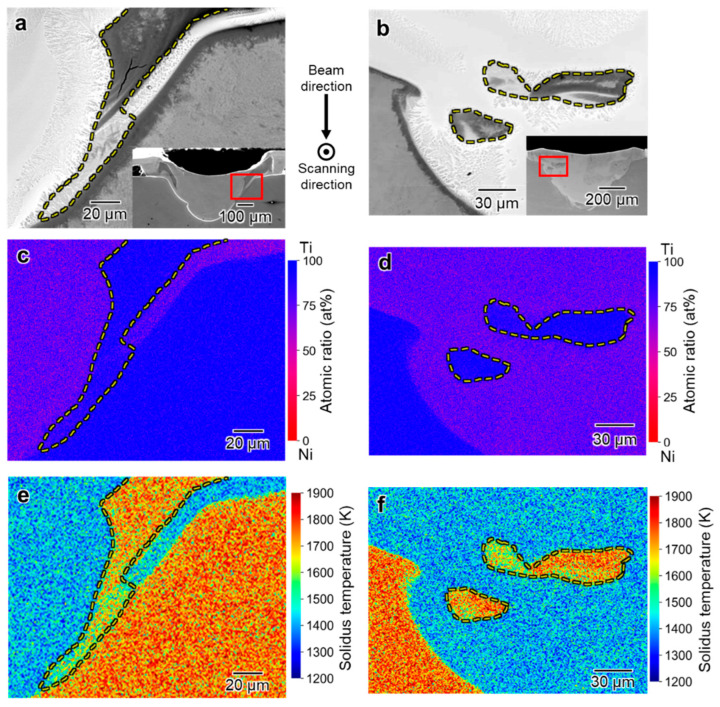
(**a**,**b**) Cross-sectional BSE images of the melt track formed with EB irradiation at *P* = 300 W and 600 W; (**c**,**d**) Ti and Ni atomic percentage mappings of the region in cross section in (**a**,**b**); (**e**,**f**) liquidus temperature mapping of the region indicated by the red squares in (**a**,**b**).

Figure 11a,b show cross-sectional SEM images of the melted region formed by EB irradiation with *P* = 300 and 600 W. Almost all the regions show uniform brightness and no obvious segregation is observed. However, there are brightness fluctuations near the fusion line, as shown in Figure 11a,b which are the magnified images indicated by the red rectangles in Figure 11a,b, respectively. The dendritic microstructures in diameters of 50–200 μm can be seen in the regions. Corresponding elemental distribution maps shown in Figure 11b,c indicate that the compositions of most of the regions and the dark contrast regions near the fusion lines are 72–76 at.% Ti, and 99–100 at.% Ti, respectively. The crystal structures of these regions are suggested to be Ti_2_Ni and Ti based on the Ti Ni phase diagram shown in Figure 9. 

It is notable that the dark contrast regions with 99–100 at.% Ti correspond to the irregular patterned regions in Figure 2. We suggest that the irregularly patterned regions are formed because of rapid heating conditions under the EB irradiation. In the AM of Al-Si alloys [31], the rapid heating causes the partial melting and the Si phase with the high-melting point remains even after the heating process. Figure 11e,f show the liquidus–temperature distribution of the regions near fusion lines (Figure 11a,b). The liquidus temperature of the region’s dark contrast in the SEM image indicated by yellow dashed lines is much higher than that of the surrounding regions. It is suggested that some of the Ti remained in the melting pool after the EB irradiation because of the higher melting point of pure Ti than that of the intermetallic phases. The remained Ti particles is supposed to flow to the edge of the melt track by the Marangoni convection [32] and be observed as an irregular pattern on the surface (Figure 2).

## 4. Discussion

### 4.1. Inhomogeneous Formation of Ti_2_Ni Phase under Low Beam Power Conditions 

The melted regions formed by the EB irradiation with *P* = 100 and 200 W have compositions close to that of the TiNi phase. However, the microstructure is inhomogeneous and the Ti-riched Ti_2_Ni phase is formed in the outermost layer of the melt pools, in which the Ti concentration is higher than the mean concentrations of the melt pools of 48.7 at.% Ti and 55.7 at.% Ti obtained by the EDS analyses. 

Jiang et al. [33] investigated the influence of solid–liquid diffusion on the microstructure at the Sn_58_Bi/Cu interface during welding. Their findings revealed that intermetallic compounds grew at the interface because of the diffusion of Cu from the substrate to the liquid during solidification. The abnormal microstructure formation due to the insufficient diffusion near the fusion line is also reported in the powder-bed fusion type AM process [34,35]. Moreover, research about the diffusion from the substrate during the formation of coatings or thin film has also been reported [36,37,38]. In this study, the two sides of the fusion line consisted of a mixture of Ti, Ni, and pure Ti. Immediately after the melt pool formation, Ti tended to diffuse from the substrate to the melt pool owing to the difference in Ti concentration. This led to an increase in the Ti concentration near the fusion line in the melt pool. Subsequently, Ti_2_Ni began to precipitate from the liquid phase. As a result, the approximate 10 μm thick layers of Ti_2_Ni are suggested to be formed under the low *P* irradiation conditions. 

**Figure 12:**
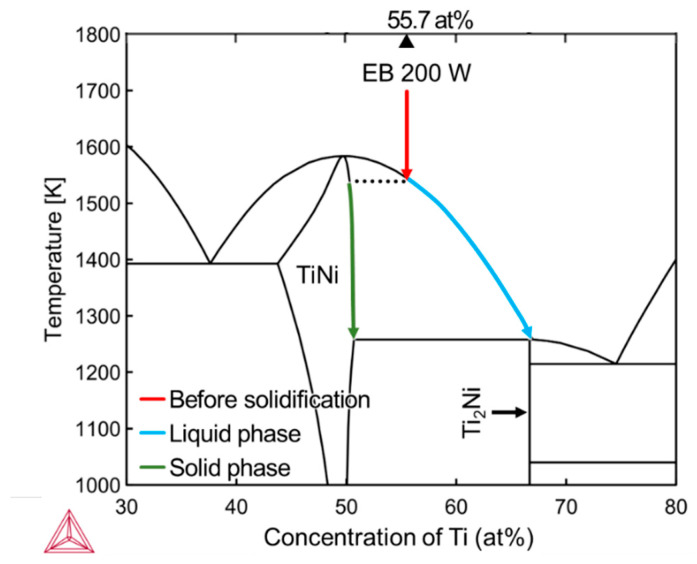
Ti-Ni binary equilibrium phase diagram calculated using the thermodynamic database for Titanium alloys (TCTI4) and the schematic of the transition during the solidification process under *P* = 200 W condition.

The formation of the Ti_2_Ni phase is also observed in the center of the melt region formed under the *P* = 200 W condition, as shown in Figure 10a. In the melting and solidification process induced by the EB irradiation, the melted liquid solidifies under a rapid cooling rate of 10^5^–10^7^ K s^−1^, which prevents the system from reaching thermodynamic equilibrium [39]. In particular, the rapid cooling rate can suppress diffusion and segregation relaxation processes, leading to a frozen-in distribution of components [40,41,42]. Figure 12 illustrates the composition transition during the solidification process in the TiNi binary system, with a Ti content of 55.70% which is the same as that in the melt track of *P* = 200 W. As the temperature of the melt pool decreased, the TiNi phase with a higher solidus temperature solidified from the liquid phase first, while the composition in the liquid phase transits along the liquid phase line toward the Ti-rich direction. Meanwhile, when the temperature decreased to the melting point of the Ti_2_Ni phase, the percentage of Ti atoms in the liquid phase also rose to 66.7%, and only then did the Ti_2_Ni phase start to precipitate out of the liquid phase. In the cross section of the melt track, the solidification process started from the fusion line, and the solid–liquid interface then advanced toward the central part. During this process, owing to the high solidification rate of EB melting, the first precipitated TiNi grains grew rapidly and occupied the surrounding area, while the remaining Ti-rich solution was pushed to the central part and finally solidified in the Ti_2_Ni phase. 

### 4.2. Strategy for Fabrication of TiNi Coating Based on the Results of This Study

This study demonstrates that the successful formation of the melt region with the almost stoichiometric composition of TiNi (Ni-50 at.% Ti) can be achieved by subjecting the Ni-plating layer on the Ti substrate to the EB scanning with the optimal EB irradiation conditions, resulting in melting and subsequent mixing of Ti and Ni. However, it should be noted that the local compositions of the melted regions are inhomogeneous and the inhomogeneity in the composition must be eliminated for fabricating TiNi coating with shape memory effects and pseudoelasticity. 

The mixing of pure Ni and Ti is supposed to be caused by the diffusion process. In addition, the Marangoni effect largely affects the melting and solidification behavior induced by the EB irradiation, in which the local region of a diameter of approximately 100 μm is solidified under the high-temperature gradient up to 10^8^ K m^−1^ [25]. The Marangoni convection occurs at the surface, originating from the center of the melted region and extending outward. The flow velocity of the convection is high up to 300 mm s^−1^, as revealed by experimentally validified computational thermal–fluid dynamic simulations [25], and the convection is suggested to accelerate the mixing of the melted pure Ni and Ti. However, the presence of inhomogeneity in the alloy composition is observed in this study although the high-velocity flow was supposed to occur in the melting and solidification processes by the EB irradiation. The exceptionally short time span of the rapid melting process in the single-track irradiation is suggested to be not enough to mix the pure Ni and Ti completely, and repeating melting and solidification of the same region is supposed to remove the inhomogeneity in the local compositions. Therefore, it is proposed that a more uniform TiNi layer can be achieved through multi-track melting under appropriate conditions. In addition, confirming the phase composition in the specimen using multiple methods including EDS and XRD allows us to investigate the effect of experimental conditions on the generation of TiNi phases and other intermetallic phases. These experiments are currently underway.

## 5. Conclusions

Ni-plated CP-Ti substrates were irradiated with the EB in six sets of parameters to obtain TiNi alloy. We observed the surface and cross section of the melt track after irradiation and analyzed it using composition analysis. The melt solidification behavior of the melt tracks was also discussed to reveal the mechanism of the formation of the microstructure. The key findings and conclusions are summarized as follows:

- EB scanning of CP-Ti substrates plated with Ni yields a dense and porosity-free microstructure within the formed melt track, exhibiting superior uniformity when compared to thermal spray coatings. The depth of the melt track is influenced by the intensity of the EB and can reach a range from 100 μm to 300 μm.
- Under optimal experimental conditions for single-track scanning, the melt track attains an elemental composition close to Ni-50 at.% Ti, which represents the stoichiometric chemical composition of the TiNi alloy. In this study, the optimal condition is an Ni-plating thickness of 44 μm, EB scanning speed of 100 mm s^−1^, and EB power of 100 W.
- There is a good homogeneity of the elemental composition in the melt track under optimal scanning experimental conditions. This can be attributed to the intense local heating caused by EB irradiation, resulting in a significant temperature gradient at the free surface. Consequently, the enhanced Marangoni convection vigorously stirs the liquid within the melt pool.
- EB Multi-Track Scanning emerges as a highly efficient technique for fabricating TiNi Self-Healing Shape Memory Coatings through the fusion of an Ni plate onto CP-Ti. This method holds the potential in achieving the desired outcome.

## Figures and Tables

**Table 1:** Composition of Ni-plating solution and plating parameters [22].

	Name	Value
Composition	NiCl_2_·6H_2_O	0.10–0.13 mol/L
	Ni(SO_3_NH_2_)_2_	1.70–1.79 mol/L
	H_3_BO_3_	0.49–0.73 mol/L
Parameters	Temperature	50–60 °C
	Cathodic current density	2.5–3.0 A/dm^2^
	pH	3.0–5.0

**Table 2:** Compositions of the selected area from melt tracks in Figure 2.

Selected Area	Composition (at.%)	
	Ti	Ni
A	99.80	0.20
B	100.00	0
C	98.22	1.88

## Data Availability

Not applicable.

## Supplementary Materials

The following supporting information can be downloaded at: https://www.mdpi.com/article/10.3390/ma16155449/s1, Figure S1. EDS spectra obtained at irregular pattern on the surface of melt track at *P* = (a) 400 W (area A in Figure 2), (b) 500 W (area B in Figure 2), and (c) 600 W (area C in Figure 2).
