# Supplementary material for: Fusion of Ni Plating on CP-Titanium by Electron Beam Single-Track Scanning: Toward a New Approach for Fabricating TiNi Self-Healing Shape Memory Coating
Source: Materials (Basel). 2023 Aug 3;16(15):5449. doi: 10.3390/ma16155449 (PMC10419621; doi:10.3390/ma16155449)
Supplement: Supplementary file 1 [file materials-16-05449-s001.zip › materials-2490269-supplementary.pdf]

Supplementary

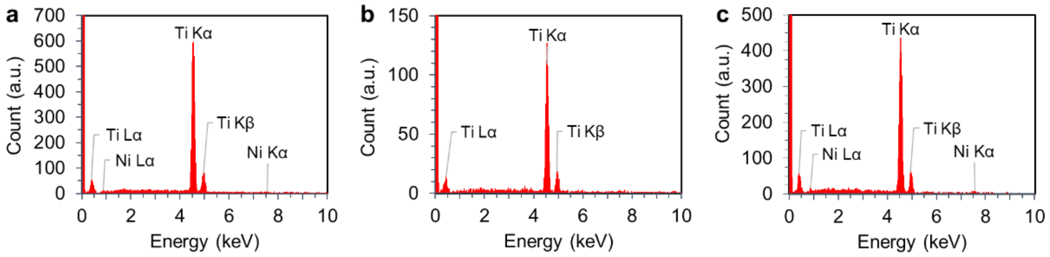

Figure S1. EDS spectra obtained at irregular pattern on the surface of melt track at *P* = (a) 400 W (area A in Figure 2), (b) 500 W (area B in Figure 2) and (c) 600 W (area C in Figure 2).
